# Temporal Profile of Endogenous Anatomical Repair and Functional Recovery following Spinal Cord Injury in Adult Zebrafish

**DOI:** 10.1371/journal.pone.0105857

**Published:** 2014-08-26

**Authors:** Katarina Vajn, Denis Suler, Jeffery A. Plunkett, Martin Oudega

**Affiliations:** 1 Department of Physical Medicine and Rehabilitation, University of Pittsburgh School of Medicine, Pittsburgh, Pennsylvania, United States of America; 2 School of Science Technology and Engineering Management, St. Thomas University, Miami Gardens, Florida, United States of America; 3 Department of Bioengineering, University of Pittsburgh School of Medicine, Pittsburgh, Pennsylvania, United States of America; 4 Department of Neurobiology, University of Pittsburgh School of Medicine, Pittsburgh, Pennsylvania, United States of America; Hertie Institute for Clinical Brain Research, University of Tuebingen, Germany

## Abstract

Regenerated cerebrospinal axons are considered to be involved in the spontaneous recovery of swimming ability following a spinal cord injury in adult zebrafish. We employed behavioral analysis, neuronal tracing, and immunocytochemistry to determine the exact temporal relationship between swimming ability and regenerated cerebrospinal axon number in adult zebrafish with a complete spinal cord transection. Between two and eight weeks post-lesion, swimming gradually improved to 44% of sham-injured zebrafish. Neurons within the reticular formation, magnocellular octaval nucleus, and nucleus of the medial longitudinal fascicle grew their axon across and at least four millimeters beyond the lesion. The largest increases in swimming ability and number of regenerated cerebrospinal axons were observed between two and four weeks post-lesion. Regression analyses revealed a significant correlation between swimming ability and the number of regenerated axons. Our results indicate the involvement of cerebrospinal axons in swimming recovery after spinal cord injury in adult zebrafish.

## Introduction

Adult zebrafish (*Danio rerio*) spontaneously recover coordinated swimming function after spinal cord injury [1]–[7]. This recovery is partial after a complete transection [1], [3]–[6] and nearly full after a crush of the spinal cord [7], demonstrating the restorative potential of the central nervous system in adult zebrafish.

After an injury to the spinal cord in adult zebrafish, tissue forms to span the lesion site [5], [7]. Ependymoradial glial cells have a central role in the formation and functioning of this tissue [5], [7], which serves as a bridge for cerebrospinal axons that grow into the caudal spinal cord [2], [4]–[9]. The reticular formation (RT), nucleus of the medial longitudinal fascicle (NMLF), and magnocellular octaval nucleus (MaON) are most regenerative with 30–50% of their neurons growing their axon across and beyond the lesion site [9].

We performed quantitative longitudinal analyses of spontaneous swimming restoration, tissue formation, and axonal regeneration in adult wild-type zebrafish after a complete spinal cord transection. Regression analysis was used to determine the relationship between swimming and regenerating cerebrospinal axons.

## Materials and Methods

### Animals

Adult zebrafish (*Danio rerio*; male or female wild-type AB* strain, 4–6 months old; <2.5 cm body length) were raised and kept on a 14 h light and 10 h dark cycle in 2.5-liter fish tanks in a double-barrier zebrafish facility with controlled air flow and room and water temperature (28.5°C). The experimental procedures were approved by the Institutional Animal Care and Use Committee at the University of Pittsburgh School of Medicine.

### Spinal cord transection and maintenance

**Figure 1:**
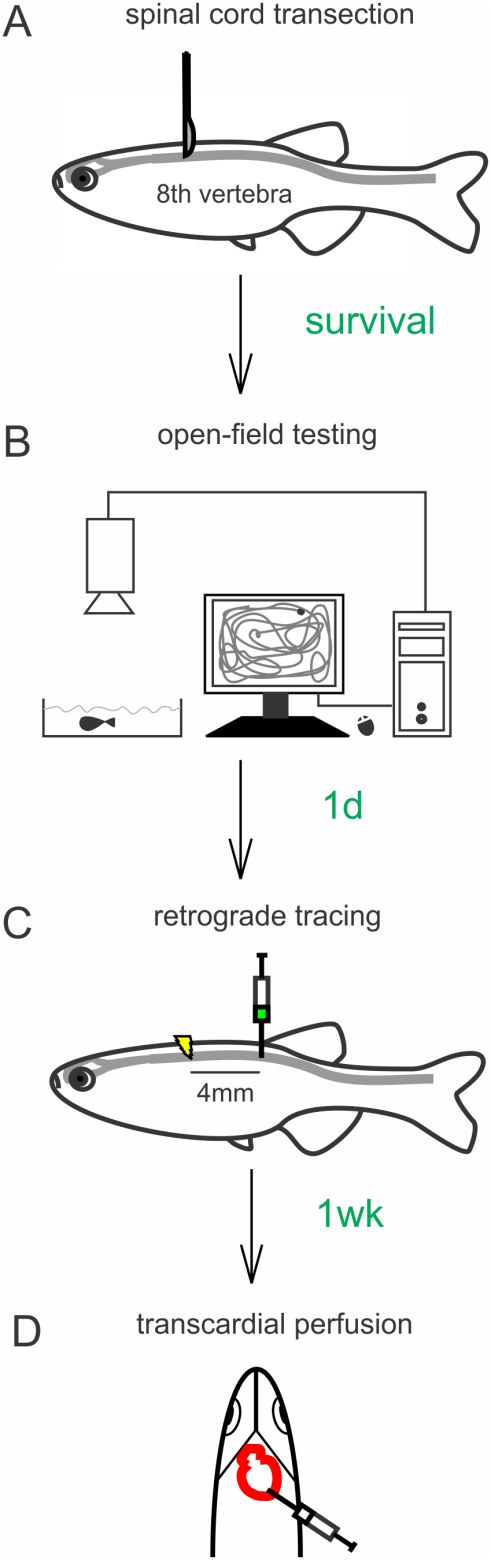
Schematic representation of the: (A) spinal cord transection, (B) open-field testing system, (C) retrograde tracing procedure, (D) transcardial perfusion. The spinal cord was cut between 8^th^ and 9^th^ vertebra. At different time points after transection (“survival”), average distance swam was measured for each fish using an open-field testing system. One day after swimming assessment Fluoroemerald was injected 4 mm caudally from the transection site. Unlesioned zebrafish were traced at the same level as lesioned zebrafish. A week after tracing, zebrafish were transcardially perfused and tissue collected for analysis. 1 d, 1 day; 1 wk, 1 week.

The zebrafish were anesthetized by immersion in 0.033% aminobenzoic acid ethylmethylester (Tricaine; Argent Laboratories, Redmond, WA) in phosphate-buffered saline (PBS; 0.1 M, pH 7.4) at 28.5°C for about 2 min. After removal of 2–3 scales at the left side at the mid-point between the brainstem-spinal cord junction and the rostral aspect of the dorsal fin, which corresponds with the eighth vertebra level (Fig. 1A), a 3 mm-long longitudinal incision was made and the muscles bluntly and gently retracted to provide access to the spinal column. With a sterilized surgical microknife (model 10055-12; Fine Science Tools, Foster City, CA) the spinal cord was completely transected between the eighth and ninth vertebra. Then, muscle tissue was cautiously maneuvered back in place and the wound closed with surgical glue (Histoacryl Blue; TissueSeal LLC, Ann Arbor, MI). The zebrafish were recovering in water at 28.5°C and, thereafter, kept individually in 2.5-liter fish tanks with Dura-Cross zebrafish breeding tank slotted inserts (Dynalon Labware, Rochester, NY) for the length of the experiment. The zebrafish were kept in the dark for the first 72 h without feeding and water change. Fungal infections were prevented with stabilized chlorine oxides (Maroxy; Sergeant’s Pet Care Products, Inc., Omaha, NE; 66 µL/L). After transection each zebrafish was randomly assigned to a particular time point group (“survival”, Fig. 1), the average swimming distance was assessed followed by retrograde tracing the next day and fixation one week later (Fig. 1).

### Behavioral testing

Swimming ability was assessed using an open-field tracking system (Ethovision XT 9.0; Noldus Information Technology Inc., Leesburg, VA) at two, four, six and eight weeks post-lesion and in unlesioned zebrafish (Fig. 1B). The zebrafish were acclimated to the testing room for 1 h prior to the testing. During the assessments, zebrafish were kept in a 26×15.5×5 cm tank with water (28.5°C) under constant lighting conditions. For each zebrafish at each of the time points, the swimming tracks were recorded and the average distance determined in two five-minute trials one hour apart.

### Neuronal tracing

Zebrafish were anesthetized as described above and the spinal cord exposed 4 mm caudal to the transection. A capillary pulled from R-6 custom glass tubing (inner diameter: 0.2 mm; Drummond Scientific Company, Broomall, PA) was prefilled with 3% Fluoroemerald (FE, Life Technologies, Grand Island, NY) in MilliQ water, attached to a Pneumatic Picopump PV-820 (World Precision Instruments Inc., Sarasota, FL), and the tip gently inserted in the spinal cord 4 mm caudally from the transection site (Fig. 1C). Then, 15 nl fluoroemerald was injected at two (n = 20), four (n = 33), six (n = 34), and eight (n = 35) weeks post-transection and in controls without transection (n = 19). The zebrafish were recovered in water at 28.5°C and were kept in standard conditions with regular feeding schedule.

### Histological procedures

One week after FE injection, the zebrafish were anesthetized as described above, perfused transcardially with PBS (0.1 M, pH 7.4) using a 1 ml insulin syringe (Fig. 1D). Then, the brain and spinal column were removed and post-fixed by immersion in 4% paraformaldehyde in PBS overnight. Next, the tissues were transferred to 30% sucrose in PBS for at least 24 h and then embedded (Richard Allen NEG50; Thermo Fisher Scientific, Waltham, MA). The brain (15 µm) and the spinal cord (10 µm) were sectioned using a cryostat (CM1950; Leica Microsystems Inc., Buffalo Grove, IL) and stored at –20°C until staining and/or examination.

### Immunohistochemistry

Spinal cord sections were incubated in 1% bovine serum albumin (Sigma-Aldrich, St. Louis, MO), 5% goat serum (Invitrogen, Carlsbad, CA) and 0.1% Triton X-100 (Sigma-Aldrich, St. Louis, MO) in PBS for 2 h. Next, the sections were incubated overnight with primary rabbit polyclonal antibodies against glial fibrillary acidic protein (GFAP, 1∶200; DAKO North America Inc., Carpinteria, CA). After washing two times for 15 min in PBS primary antibody binding was detected with Alexa Fluor 546 Goat Anti-Rabbit IgG (H+L) (Life technologies, Grand Island, NY). The sections were then washed two times for 15 min, covered with glass slips in Fluorescence Mounting Medium (DAKO North America Inc., Carpinteria, CA), and stored at –20°C until examination.

### Assessment of labeled neurons

A Zeiss Axioskop inverted microscope (Carl Zeiss Microscopy, LLC; Thornwood, NY) was used to identify FE-labeled neurons in the brain. All FE-labeled neurons were counted in brain including the NMLF, MaON, and RT. The RT included the superior reticular nucleus, intermediate reticular nucleus and the inferior reticular nucleus. CorelDraw 12 software (Corel Inc., Mountain View, CA) was used to assemble final figures, which were adjusted for contrast, intensity and brightness.

### Statistical analysis

SPSS Statistics (IBM Software, Armonk, NY) was used for statistical analyses. Kolmogorov-Smirnov and Shapiro-Wilk’s tests were used to determine whether the distribution of data was normal, and Levene’s test was used to determine the homogeneity of variance. Upon determining the non-normality of distribution and the non-homogeneity of variance, statistical significance was determined using the non-parametric Kruskal-Wallis test for multiple comparisons followed by Mann-Whitney-U tests for single comparisons with Bonferroni adjustment for the 10–15 comparisons that were conducted. Differences were considered significant with p≤0.005 (10 comparisons) or p≤0.003 (15 comparisons). Because the data was non-parametric, we used the median as a measure of central tendency with the variability given as a 95% confidence interval of the median. The cumulative frequency was calculated and plotted for the behavioral data to illustrate the non-normality/normality of distribution in individual groups. The neuron counts and average distance swam were ranked for each zebrafish in a group and Spearman’s rho test was used to determine correlations between these two variables. Correlation was considered significant with p<0.05.

## Results

### Swimming ability gradually increases after spinal cord transection

One day post-lesion the injured zebrafish mainly stayed still at the bottom of the tank and swam only for short periods of time using their pectoral fins (Movie S1). At 2 weeks post-lesion some fish exhibited smooth movements of the tail fin (Movie S2), but most still swam using their pectoral fins (Movie S3). Over the ensuing weeks, most of the injured fish demonstrated increasingly smooth coordinated movements of the tail fin (Movie S4) which resembled those of normal (unlesioned) fish (Movie S5).

**Figure 2:**
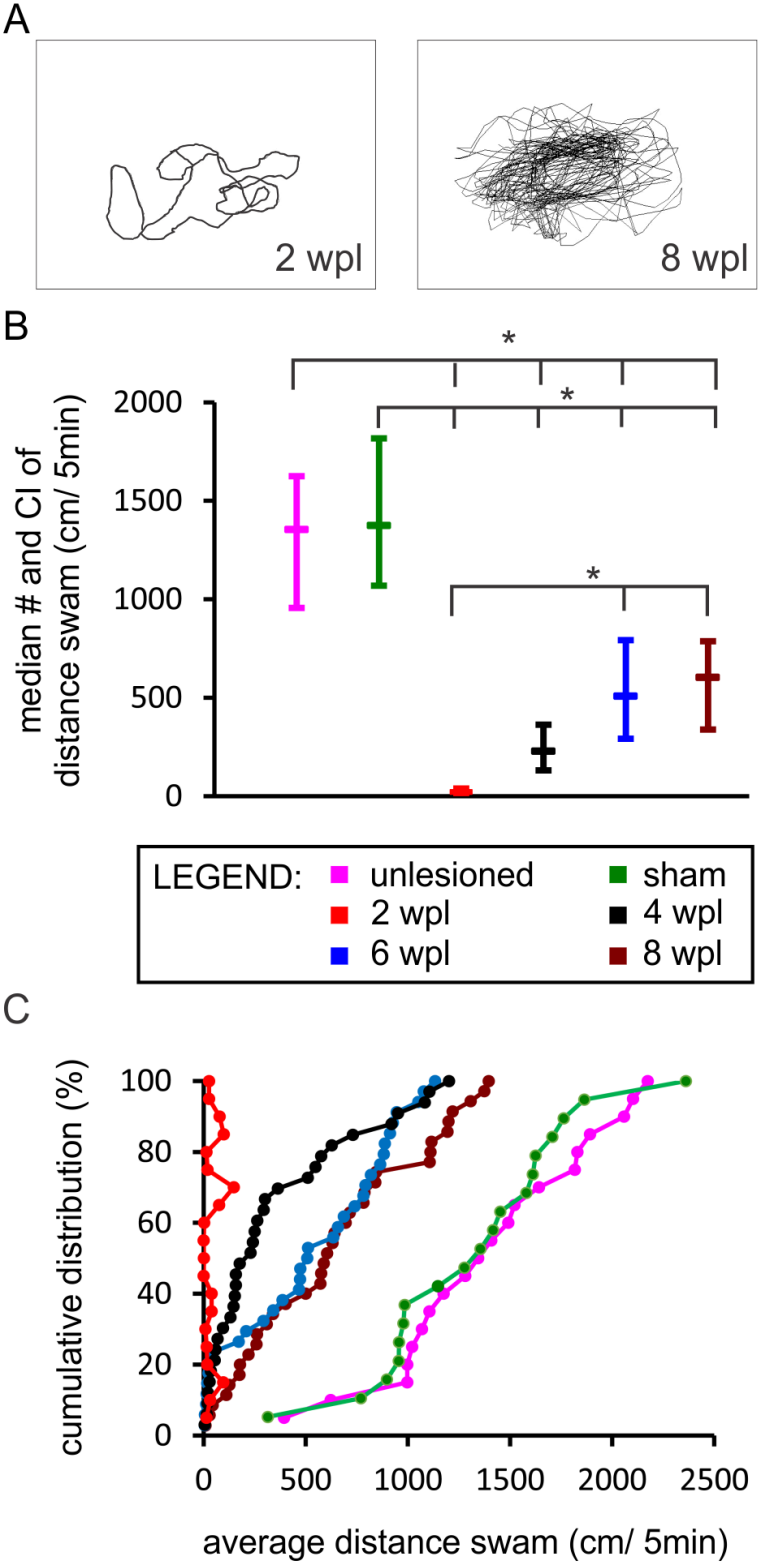
Swimming ability improves over a period of eight weeks after a complete spinal cord transection in adult zebrafish. (A) Examples of swimming path recorded in a five-min period at two- (“2 wpl”) and eight (“8 wpl”) weeks post-lesion. (B) Boxplot diagram demonstrating the median distance swam and confidence interval of the median during the five-min period. Asterisks indicate significant differences between groups (Kruskal-Wallis test, followed by Mann-Whitney U test with Bonferroni correction, p significant if ≤0.003). Two weeks post-lesion group is significantly different from all other groups. Normal (unlesioned) group is significantly different from all other groups. (C) A cumulative distribution plot illustrating the differences in the distribution of data between individual groups. (unlesioned (n = 19), sham-injured (n = 20), 2 wpl (n = 20), 4 wpl (n = 33), 6 wpl (n = 34), 8 wpl (n = 35).

With an open-field tracking system swimming was recorded and the distance measured (for the original data see Supporting Information S1). Examples of raw tracking data are provided in figure 2A. The median total swimming distance over a five min period increased from 22 cm at two (95% CI [13.45, 38.84], n = 20), 229 cm at four (95% CI [131.23, 363.13], n = 33), 509 cm at six (95% CI [292.07, 792.60], n = 34), and 604 cm at eight (95% CI [338.69, 787.15], n = 35) weeks post-lesion (Fig. 2B). Compared with two weeks, swimming distance improved 10-fold at four, 23-fold at six, and 27-fold at eight weeks post-lesion. At each post-lesion time point, injured zebrafish swam less than uninjured zebrafish (median: 1355 cm; 95% CI [956.37, 1625.12], n = 19) (Fig. 2B) and sham-injured fish (median: 1376 cm; 95% CI [1069.26, 1817.34], n = 20) (Fig. 2B). A cumulative distribution plot (Fig. 2C) illustrates the distribution of data at six- and eight- weeks post-lesion approaching the distribution observed in the sham and unlesioned group. At eight weeks post-lesion, swimming ability had recovered to 44% of the sham-injured fish swimming ability. Our data show that swimming ability gradually recovers after a spinal cord transection with the largest relative improvement between two and four weeks post-lesion.

### Generation of new tissue at the spinal cord transection site

**Figure 3:**
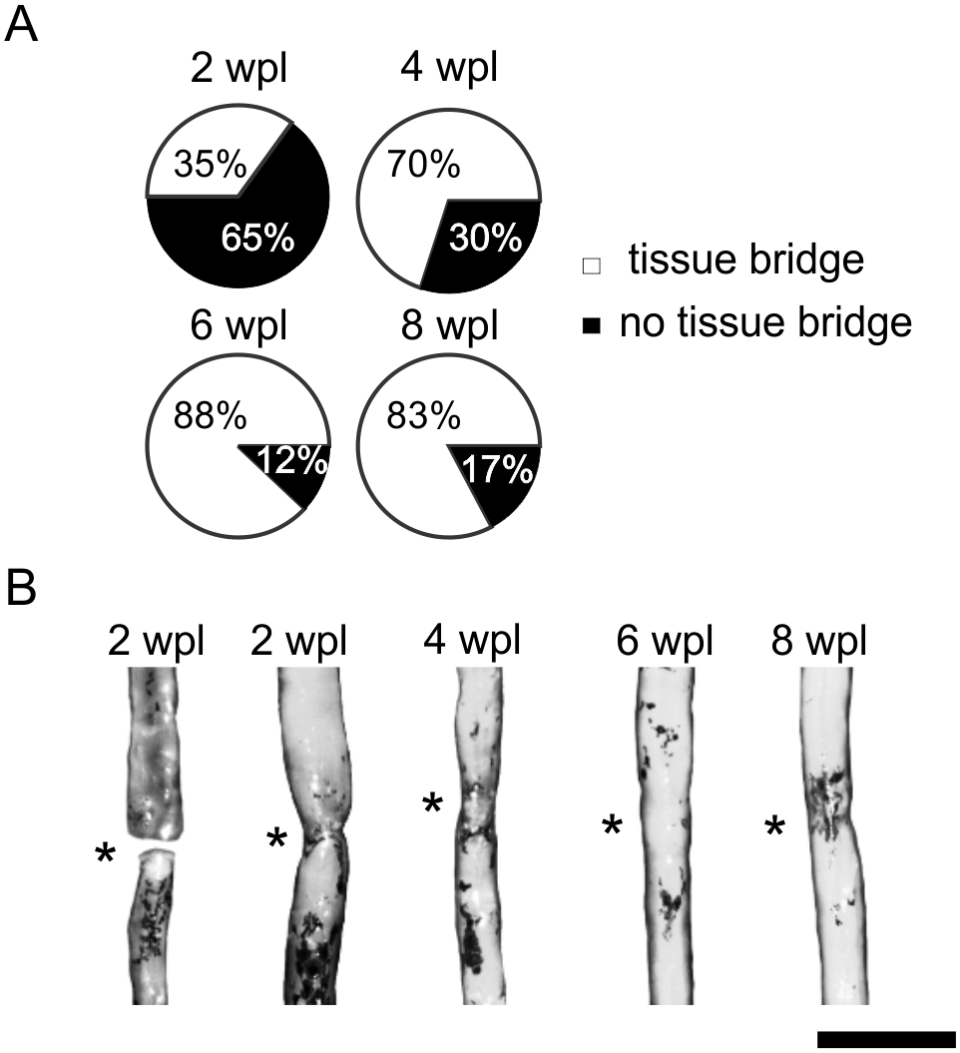
New tissue forms within the spinal cord transection site in adult zebrafish. (A) Percentage of zebrafish with (white) and without (black) a tissue bridge at the different time points post-lesion. (B) Photographs of dissected spinal cord showing the new tissue in the transection site (asterisks) and bridging the spinal cord stumps. Bar = 1 mm.

**Figure 4:**
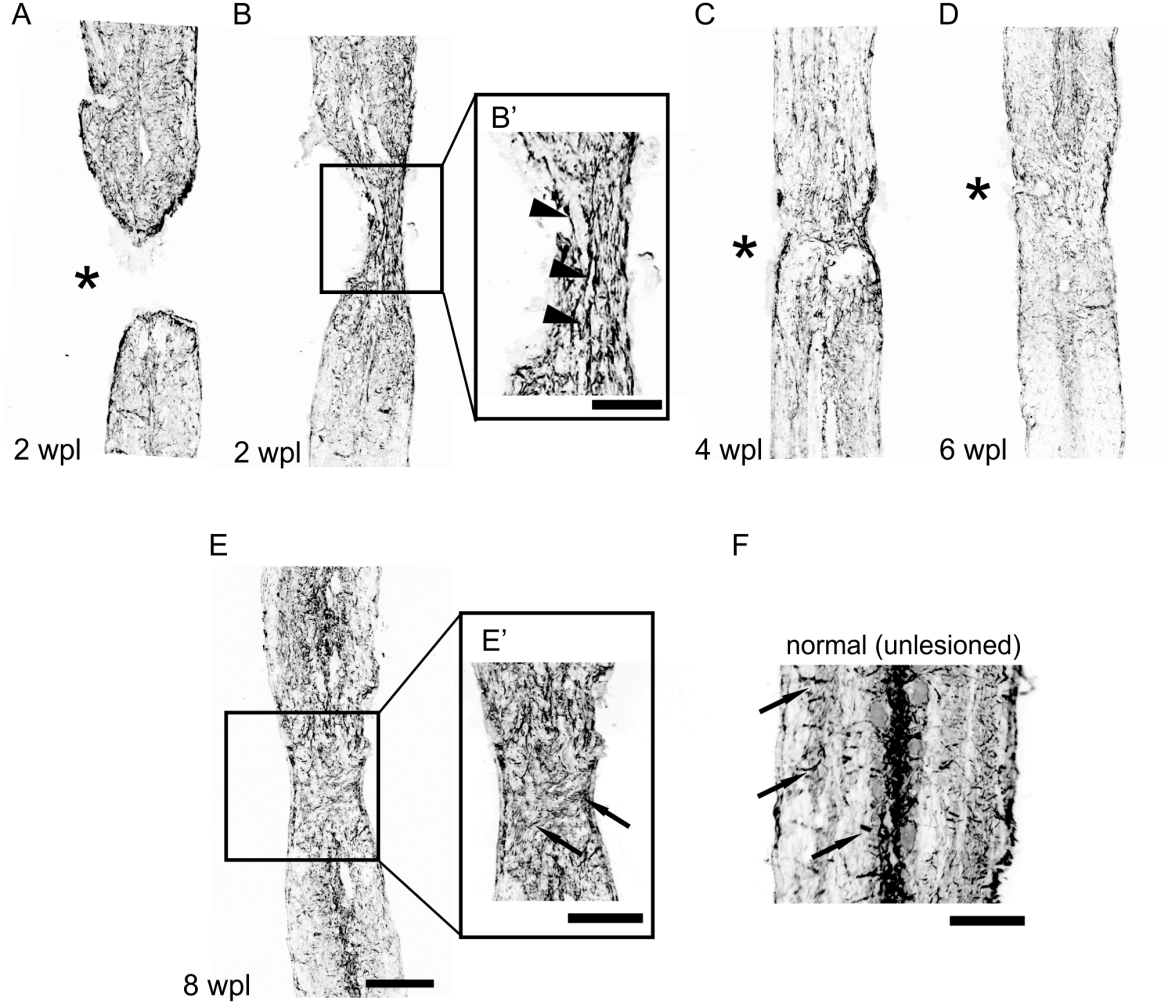
GFAP-positive cells form a tissue bridge at the spinal cord transection site. (A) Distribution and orientation of GFAP-positive cells and processes at two weeks post-lesion in zebrafish without the tissue bridge. Distribution and orientation of GFAP-positive cells and processes in zebrafish with the tissue bridge at two weeks post-lesion (B), four weeks post-lesion (C), six weeks post-lesion (D) and eight weeks post-lesion (E). (B’) Enlarged view of the newly formed tissue from box in B. Arrowheads point at the longitudinally oriented GFAP-positive processes. (E’) Enlarged view of the newly formed tissue from box in E. (F) Distribution of GFAP-positive cells and processes in normal (unlesioned) tissue. Arrows point at the radially oriented GFAP-positive processes. Transection site is denoted with asterisks. Bar = 100 µm in A–E and 50 µm in B’, E’, F.

After complete transection, the spinal cord stumps slightly retracted. In time, new tissue was generated in the lesion forming a bridge between the rostral and caudal spinal cord. The lesion was bridged by new tissue in 35% of the injured zebrafish at two weeks (Figs. 3A, 3B). An example of the spinal cord at two weeks post-lesion in which a tissue bridge is not yet present is provided in figure 4A. The advent of the tissue bridge correlated with the onset of swimming recovery. A tissue bridge was found in 70% of the zebrafish at four weeks, 88% at six weeks, and 83% at eight weeks post-lesion (Figs. 3A, 3B). At six weeks and later, the diameter of the tissue bridge was similar to the spinal cord at the comparable level in unlesioned zebrafish (Fig. 3B). GFAP-positive cells with longitudinally oriented processes appeared in the newly formed tissue during the second week post-lesion (Figs. 4B, 4B’). The GFAP pattern gradually developed into that seen in adult unlesioned zebrafish (Figs. 4C–F). The results show that new tissue gradually forms between the spinal cord stumps starting during the second week post-transection facilitating the onset of coordinated swimming recovery.

### Axons regenerate beyond a complete spinal cord transection

**Figure 5:**
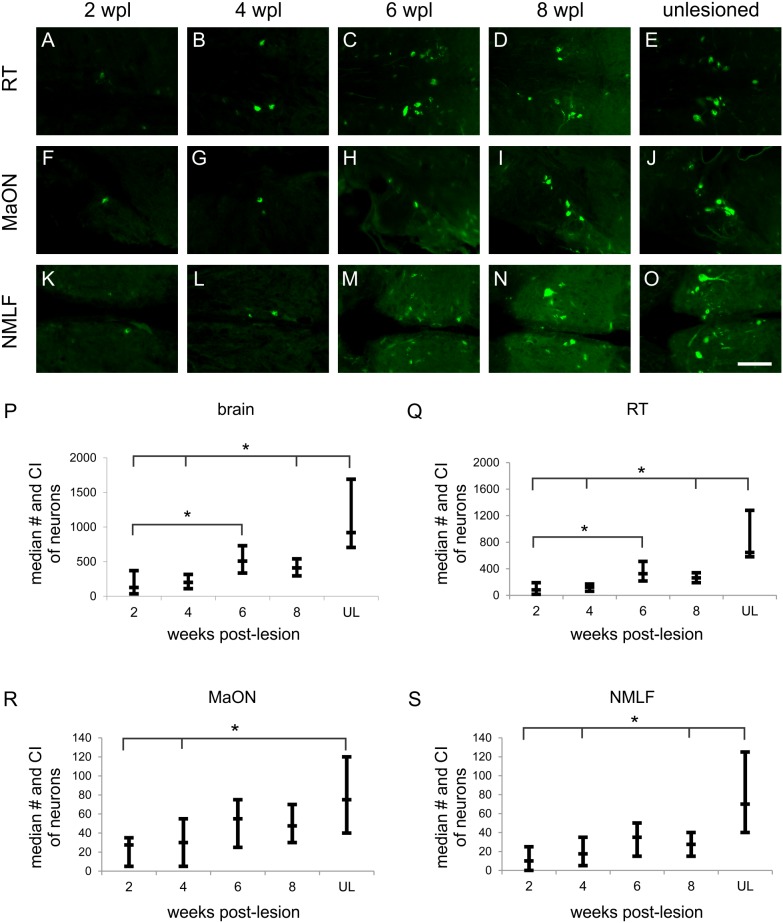
The number of cerebrospinal axons regenerated into the caudal spinal cord increases in time after the lesion. (A–E) Retrogradely labeled neurons in RT (A–E), MaON (F–J), and NMLF (K–O) at two (A, F, K, respectively), four (B, G, L, respectively), six (C, H, M, respectively), and eight (D, I, N, respectively) weeks post-lesion and in unlesioned zebrafish (E, J, O, respectively). Boxplot diagrams demonstrating the median number (and confidence interval of median) of FE-labeled neurons in zebrafish at two, four, six, and eight weeks post-lesion and in unlesioned zebrafish in the whole brain (P), RT (Q), MaON (R), and NMLF (S). Asterisks indicate significant differences between groups (Kruskal-Wallis test, followed by Mann-Whitney U test with Bonferroni correction, p significant if ≤0.005). The numbers of total FE-positive neurons and FE-positive neurons in RT are significantly different between two weeks post-lesion and six weeks post-lesion. The number of FE-positive neurons in normal (unlesioned) group is not significantly different from six weeks post-lesion in the whole brain and in each of the examined nuclei. 2 wpl (n = 20); 4 wpl (n = 21); 6 wpl (n = 20); 8 wpl (n = 20), unlesioned (n = 19). Bar = 100 µm in A–O.

To determine axonal regeneration across and beyond the newly formed tissue bridge, we used FE to retrogradely label neurons with an axon projecting into the caudal spinal cord. FE-labeled neurons were consistently found in the RT (Figs. 5A–D), MaON (Figs. 5F–I), and NMLF (Figs. 5K–N) at all times post-lesion. The presence of labeled neurons in the RT, MaON, and NMLF in unlesioned age-matched zebrafish is shown in figures 5E, J, and O, respectively. Lesioned fish also had FE-labeled neurons in the periventricular nucleus of posterior tuberculum, nucleus of the lateral lemniscus, anterior octaval nucleus, descending octaval nucleus, inferior raphe, and Mauthner neurons. The number of FE-labeled neurons in these nuclei was highly variable between zebrafish; we counted these together as ‘other nuclei’. At eight weeks post-lesion, the mean total number of the FE-labeled neurons in these ‘other nuclei’ was 17% of that in the whole brain.

Quantification revealed that from two weeks post-lesion on, the number of FE-positive neurons gradually increased in the RT to a median of 263 (95% CI [190, 340], n = 20; Fig. 5Q), in the MaON to 48 (95% CI [30, 70], n = 20; Fig. 5R), and in the NMLF to 28 (95% CI [15, 40], n = 20; Fig. 5S) at eight weeks post-lesion. At this time point, the number of FE-labeled neurons in the whole brain had gradually increased to a median of 410 (95% CI [295, 540], n = 20); Fig. 5P). The total number of FE-labeled neurons was similar at six and eight weeks post-lesion. The total number of FE-labeled neurons at six weeks post-lesion was not significantly different than that in unlesioned (normal) adult zebrafish (Fig. 5P). At eight weeks, the number of labeled neurons compared with unlesioned zebrafish was 41% in the RT, 64% in the MaON, 40% in the NMLF, and 45% in the whole brain. The data show that cerebrospinal axons start regenerating across and beyond the newly formed tissue bridge in the second week post-lesion.

### Axonal regeneration correlates with swimming capability

**Figure 6:**
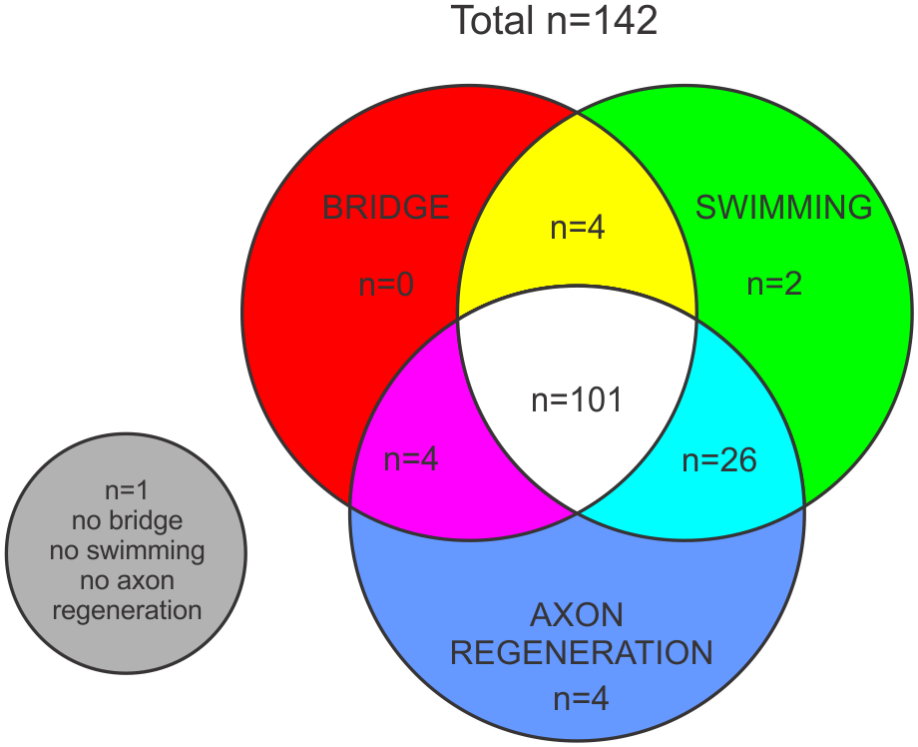
Venn diagram denoting the number of zebrafish belonging to each set. Zebrafish that had a nervous-like tissue bridge (set “BRIDGE”), zebrafish that swam ≥10 cm/5 min (set “SWIMMING”), and zebrafish that had ≥1 FE-positive neuron in brain (set “AXON REGENERATION”).

In order to test the hypothesis that swimming recovery is influenced by tissue bridge formation and cerebrospinal axon regeneration, we examined each of the analyzed zebrafish (n = 142) for presence of tissue bridge at dissection, average distance swam and the presence of FE-labeled neurons in the brain (Fig. 6). Since the transection has been performed at the 8th vertebra, the zebrafish have been able to use their pectoral fins and swim some distance right after the transection (Movie S1). Hence, for further analysis we defined all the zebrafish that swam ≤10 cm/5 min as ‘not swimming’ (n = 9). We found that most of the fish examined (n = 101) had a tissue bridge at dissection, swam ≥10 cm/5 min and had at least 1 FE-labeled neuron in the brain. About 23% of zebrafish (n = 32) swam some distance although they had either no tissue bridge (n = 28), no FE-labeled neurons in the brain (n = 6) or both (n = 2).

**Figure 7:**
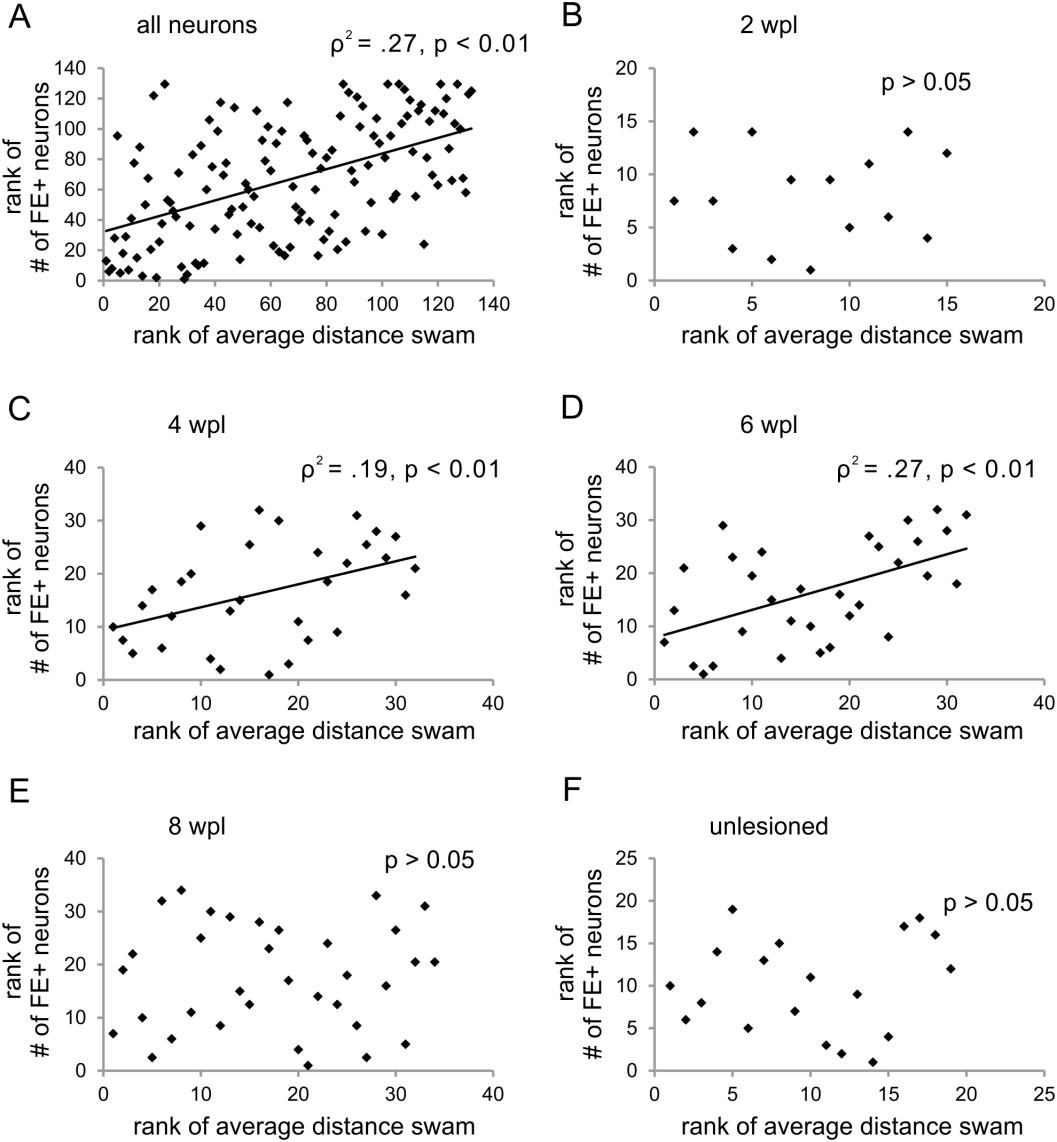
Cerebrospinal axon regeneration is correlated with swimming ability. (A) Rank of number of FE-positive neurons in the whole brain is correlated with the rank of average distance swam in all analyzed zebrafish (n = 132) and, specifically, at (C) four (n = 32) and (D) six (n = 32) weeks post-lesion. No correlation was observed at (B) two (n = 15) and (E) eight (n = 34) weeks post-lesion and in (F) normal (unlesioned) zebrafish (n = 19). Spearman’s rho correlation, differences considered significant with p<0.05.

**Figure 8:**
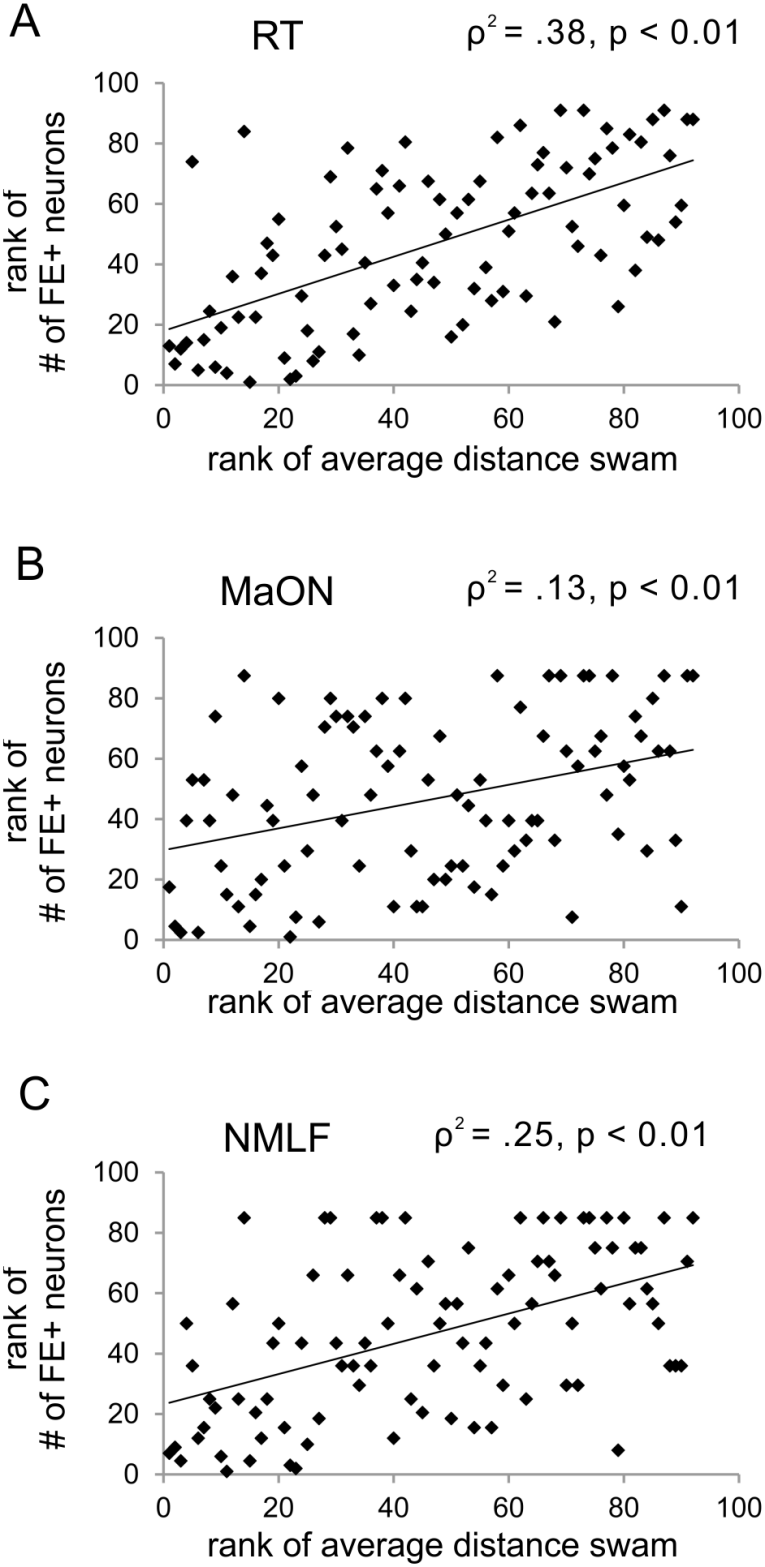
Axonal regeneration from specific brain nuclei is correlated with swimming ability. In all analyzed zebrafish, the rank of average distance swam is correlated with the rank of number of FE-positive neurons in the (A) RT, (B) MaON, and the (C) NMLF. Spearman’s rho correlation, differences considered significant with p<0.05, n = 92.

Next, after exclusion of the not swimming zebrafish we performed regression analyses to determine the exact relationship between the number of traced neurons and average swimming distance. Regression analyses revealed that, taking all zebrafish and all time points in account, the number of FE-labeled neurons correlated with swimming ability (ρ^2^ = .27, p (one-tailed) <0.01; n = 132; Fig. 7A). A more detailed analysis showed that these two parameters correlated at four (ρ^2^ = .19, p (one-tailed) <0.01; n = 32; Fig. 7C) and six (ρ^2^ = .27, p (one-tailed) <0.01; n = 32; Fig. 7D) weeks post-lesion. A correlation between swimming and FE-labeled neurons was not found at two (Fig. 7B) and eight (Fig. 7E) weeks post-lesion or in unlesioned adult zebrafish (Fig. 7F). Investigating the individual nuclei, we found that swimming ability correlated with the number of FE-positive neurons in the RT (ρ^2^ = .38, p (one-tailed) <0.01, n = 92; Fig. 8A), MaON (ρ^2^ = .13, p (one-tailed) <0.01, n = 92, Fig. 8B), and NMLF (ρ^2^ = .25, p (one-tailed) <0.01, n = 92; Fig. 8C).

## Discussion

Our data showed that adult zebrafish gradually regain their ability to generate coordinated swimming movements between two and eight weeks after a complete spinal cord transection. The largest relative improvement in swimming was found between the second and fourth week post-injury. Recovery was incomplete reaching a maximum of 44% of the swimming ability of sham-injured age-matched zebrafish at eight weeks post-injury. These data on the recovery of swimming after a complete spinal cord transection were largely in agreement with previous studies [1], [4]. The average post-lesion swimming distance in our study was shorter than that reported at comparable time-points which may account for the lower maximum recovery of 44% vs. 57% [1] or 78% [4]. The lack of an adaptation period prior to measuring swimming distance in our study may explain these overall shorter distances [4]. Also, the use of a shorter time interval between trials [1] may have contributed to the observed difference in swimming distances.

We demonstrated that cerebrospinal axons extend beyond the transection site starting from the second week post-injury. These axons derived mostly from existing brainstem neurons because a possible contribution of newly generated brainstem neurons is either small [9] or absent [6]. The largest relative growth was found between the second and fourth week post-injury. At six weeks, the total number of regenerated cerebrospinal axons reached its maximum at 55% of normal projection in unlesioned age-matched zebrafish. Among the examined nuclei, the RT contained the largest number of neurons projecting beyond the lesion and the MaON exhibited the largest relative regenerative capacity with 64% of its normal projection in unlesioned zebrafish.

This is the first quantitative study of the regenerative response of cerebrospinal axons during the first weeks after spinal cord injury in adult zebrafish. Our data is in agreement with the finding that neurons in the RT, MaON, and NMLF are most regenerative in adult zebrafish after spinal cord injury [9]. The percentage of regenerating neurons at six weeks post-lesion is largely similar between our and this previous study [9]. However, the total number of retrogradely-labeled (regenerating) neurons is higher in the present study, which could be due to the use of different retrograde tracers; Fluoroemerald in the present study and biocytin in the previous study [9]. An alternative explanation may be that in time regenerated axons are pruned from the caudal spinal cord which would explain lower overall number of back-filled neurons with tracer injections at later time points. Indeed, our finding of lower numbers of labeled neurons in the whole brain and in the examined nuclei, at 8 weeks as compared to 6 weeks, supports such a pruning mechanism.

Our data showed that, with time, newly formed tissue bridged the spinal cord stumps in an increasing number of zebrafish, indicating variability in the ability to generate new tissue. From six weeks on, this bridge had a similar diameter and appeared essentially similar as the unlesioned spinal cord at the same spinal cord segment. We found that in the new tissue, an early mostly longitudinal pattern of GFAP-positive processes developed gradually into a more typical radial pattern, which is congruent with previously published data [5]. The consecutive longitudinal and radial pattern of GFAP-positive processes reflected the initial presence of elongated bipolar glial cells bridging the spinal cord stumps and the final presence of radial glia, a hallmark of normal unlesioned spinal cord tissue, which was apparent around week six post-lesion when a central canal had been reconstructed [5]. Interestingly, we found the tissue bridge permissive to axon growth starting around two weeks post-lesion, which was before its architecture had finalized in that typical for unlesioned nervous tissue. In fact, the longitudinal GFAP-positive processes facilitated axon regeneration across the bridge [5]. It is also possible that radially oriented GFAP-processes impeded axon regeneration, which could explain the relative lower regenerative responses at later post-lesion time points. Altogether, it is likely that the development of the new tissue into mature-appearing nervous tissue at least in part determined the magnitude of the axon growth process and thus of the overall swimming recovery. Cellular or molecular manipulation of this maturation process may therefore be used as a tool to influence the overall repair and recovery after spinal cord injury.

The onset of coordinated swimming was noticed around two weeks post-lesion which correlates with the time when new tissue was found spanning the lesion and when the first cerebrospinal axons were found in the caudal spinal cord. The temporal profiles of swimming recovery and axon regeneration were largely similar with a progressive increase that starts to plateau after six weeks post-lesion. For both swimming and axon regeneration around 50% of the maximum was reached at four weeks post-lesion. This means that in most fish at this time point, at least half of the axons were still actively regenerating.

Taking all post-lesion time points into account, regression analysis confirmed the presence of a significant correlation between swimming distance and cerebrospinal axon number. When specific post-lesion time points were analyzed, the correlation between swimming and cerebrospinal axon number was present at four and six weeks, but not at two and eight weeks post-lesion. The lack of a relationship at two weeks post-lesion may reflect the relatively small number of axons that regenerated beyond the lesion at this early time point. Also, at two weeks, 65% of all zebrafish had not yet formed a complete tissue bridge between the spinal cord stumps. The lack of a relationship between swimming and axon number at eight weeks post-lesion could have been due to a pruning effect that was apparent at this time point.

Dopaminergic and serotonergic axons from neurons in diencephalic and raphe nuclei, respectively, have been implicated in swimming ability in adult zebrafish [2]. However, these nuclei project only a few axons to spinal cord and rarely regenerate axons as evidenced by Beckers *et al*. [9] and confirmed by our own data. Additionally, these types of neurons regenerate their axon over relatively short distances beyond the transection site [2]. In contrast, the significant correlation between the number of regenerated axons originating from RT, MaON and NMLF and the average swimming distance suggests that these nuclei are the most likely candidates in the descending control of locomotion. These nuclei have a large number of neurons projecting to mid-thoracic level of spinal cord and have been shown to consistently regenerate about half of that projection to at least 4 mm beyond the lesion by six weeks post-lesion.

Supporting the idea that RT, MaON and NMLF are the principal nuclei involved in the descending control of zebrafish swimming is a recent study by Kyriakatos *et al.* [10] Using a model of fictive locomotion, they investigated the origin of descending inputs to central pattern generators of the spinal cord in the adult unlesioned zebrafish. While the stimulated nuclei were not anatomically identified, the position of the electrodes in their paper indicates that they most likely stimulated reticular nuclei and/or the medial longitudinal fasciculus. Additionally, it was also recently shown that reticulospinal glutamatergic V2a neurons are involved in the excitation of spinal locomotor circuits during larval zebrafish swimming [11].

The key finding of this study is that the axons of RT, MaON and NMLF actively regenerate between the second and fourth week post-lesion. A comprehensive gene expression data on populations of regenerating vs. non-regenerating neurons, collected during this period, may provide us with key molecules involved in the axonal regeneration. The information on molecules involved in the successful axonal regeneration can further be used to create transgenic zebrafish with labeled regenerating axons to aid future functional and connectomics studies.

## Supporting Information

Movie S1 **Zebrafish swimming at one day post-lesion.**
(MP4)

Movie S2 **Zebrafish swimming at two weeks post-lesion (with smooth movements of the tail fin).**
(MP4)

Movie S3 **Zebrafish swimming at two weeks post-lesion (without smooth movements of the tail fin).**
(MP4)

Movie S4 **Zebrafish swimming at four weeks post-lesion.**
(MP4)

Movie S5 **Unlesioned zebrafish swimming.**
(MP4)

Supporting Information S1 **Original data.**
(XLSX)
